# Comparative evaluation of the health utilities index mark 3 and the short form 6D: evidence from an individual participant data meta-analysis of very preterm and very low birthweight adults

**DOI:** 10.1007/s11136-023-03344-x

**Published:** 2023-01-27

**Authors:** Corneliu Bolbocean, Peter J. Anderson, Peter Bartmann, Jeanie L. Y. Cheong, Lex W. Doyle, Dieter Wolke, Stavros Petrou

**Affiliations:** 1 Nuffield Department of Primary Care Health Sciences, University of Oxford, Radcliffe Observatory Quarter, Woodstock Road, Oxford, OX2 6GG UK; 2 School of Psychological Sciences, Turner Institute for Brain and Mental Health, Monash University, Melbourne, VIC Australia; 3 Clinical Sciences, Murdoch Children’s Research Institute, Melbourne, VIC Australia; 4 Department of Neonatology and Paediatric Intensive Care, Children’s Hospital, University Hospital Bonn, Bonn, Germany; 5 Newborn Services, Royal Women’s Hospital, Parkville, VIC Australia; 6 Department of Obstetrics and Gynaecology, University of Melbourne, Melbourne, VIC Australia; 7 Department Paediatrics, The University of Melbourne, Melbourne, VIC Australia; 8 Department of Psychology, Warwick Medical School, University of Warwick and Division of Health Sciences, Coventry, UK

**Keywords:** HUI3, SF-6D, HRQoL, Health utilities, Very preterm birth, Very low birth weight

## Abstract

**Background:**

The most appropriate preference-based health-related quality of life (HRQoL) instruments for trials or research studies that ascertain the consequences of individuals born very preterm and/or low birthweight (VP/VLBW) are not known. Agreement between the HUI3 and SF-6D multi-attribute utility measures have not been previously investigated for VP/VLBW and normal birthweight or term-born controls. This study examined the agreement between the outputs of the HUI3 and SF-6D measures among adults born VP/VLBW and normal birthweight or term born controls.

**Methods:**

We used two prospective cohorts of individuals born VP/VLBW and controls contributing to the ‘Research on European Children and Adults Born Preterm’ (RECAP) consortium which assessed HRQoL using two preference-based measures. The combined dataset of individual participant data (IPD) included 407 adult VP/VLBW survivors and 367 controls, ranging in age from 18 to 26 years. Bland–Altman plots, intra-class correlation coefficients, and generalized linear mixed models in a one-step approach were used to examine agreement between the measures.

**Results:**

There was significant discordance between the HUI3 and SF-6D multi-attribute utility measures in the VP/VLBW sample, controls, and in the combined samples. Agreement between the HUI3 and SF-6D multi-attribute utility measures was weaker in controls compared with VP/VLBW individuals.

**Conclusions and relevance:**

The HUI3 and SF-6D each provide unique information on different aspects of health status across the groups. The HUI3 better captures preterm-related changes to HRQoL in adulthood compared to SF-6D. Studies focused on measuring physical or cognitive aspects of health will likely benefit from using the HUI3 instead of the SF-6D, regardless of gestational age at birth and birthweight status.

**Supplementary Information:**

The online version contains supplementary material available at 10.1007/s11136-023-03344-x.

## Introduction

Health-related quality of life (HRQoL) is an important aid for evaluating clinical and policy interventions [1–3] and can be defined as “how well a person functions in their life and his or her perceived well-being in physical, mental, and social domains of health” [4]. Functioning refers to an individual’s ability to carry out some pre-defined activities; however, well-being is understood as an individual’s subjective feeling(s) [4]. HRQoL measures that are accompanied by preference-based value sets generate utility scores that reflect preferences for health states on a cardinal scale where 0 represents being dead and 1 represents full health [1]. Preference-based HRQoL measures are widely recommended for use in publicly funded health care systems because of their role in cost-utility analysis, which in forms reimbursement, regulatory, and pricing mechanisms [3, 5–8]. Furthermore, they are increasingly used as outcome measures in clinical trials and patient care [9–12] across a wide spectrum of conditions and environments [1–3, 13–15] partly because they are highly correlated with widely used health metrics, including morbidity, mortality, and healthcare costs [2, 3, 16]. However, there is discordance between the preference-based HRQoL measures that are recommended for use by health technology assessment agencies in different jurisdictions [17]. Guidelines for selecting preference-based HRQoL instruments for randomized trials and observational studies are lacking.

Preference-based HRQoL measures are made up of descriptive systems and accompanying valuation systems. The descriptive system defines HRQoL across a number of health states and the valuation system is a mathematical construct for scoring each possible health state described by the measure. The valuation or scoring system generates utility scores that reflect population preferences for living in a particular health state. While these utility scores are indexed on a cardinal scale where 1 indicates full health and 0 represents death, negative values are theoretically possible and represent health states considered worse than death [1–3, 18].

The choice of a preference-based HRQoL measure is a critical decision because of downstream consequences related to cost-utility analysis, its use for deriving quality-adjusted life-years (QALYs), and subsequent resource allocation decisions [19]. Thus, evidence comparing the performance of preference-based HRQoL measures is needed to justify the selection of the most appropriate assessment tool [20]. Furthermore, researchers attempting to measure health outcomes face a trade-off on whether to include a single or multiple HRQoL instruments in their studies. The latter option is not always possible because of budgetary and time constraints as well as evidence for lower completion rates when multiple instruments are used [21–23].

The Health Utilities Index Mark 3 (HUI3) and Short Form 6D (SF-6D) are two widely used preference-based HRQoL measures that are anchored on a cardinal scale (with 0 = dead and 1 = full health) and generate utility scores that reflect population preferences for health states as well as for estimating QALYs for cost-utility analysis purposes [1–3, 18]. HRQoL measures accompanied by preference-based value sets are often referred to as multi-attribute utility instruments in the literature [1–3, 18]. Scoring algorithms for these measures have been derived based on nationally representative, community-based samples from different jurisdictions, such as Canada (HUI3) and the United Kingdom (SF-6D) [2, 3].

A recent review from health technology assessment (HTA) agencies regarding the preferred choice of preference-based HRQoL measures for cost-effectiveness based decision-making identified that, out of thirty-four guidelines, twenty-one recommended either the SF-6D (*n* = 11) or HUI2 or HUI3 (*n* = 10) instruments [17]. There is limited evidence for concurrent health status assessments using both the HUI3 and SF-6D instruments. However, at the same time, there is little consensus about the head-to-head performance of preference-based HRQoL measures across psychometric criteria.

Individuals born very preterm (VP; < 32 weeks’ gestation) or at very low birthweight (VLBW; < 1500 g) are at high-risk of adverse functional, neurodevelopmental and behavioral outcomes [24–28] and their HRQoL is frequently examined because of increasing rates of preterm birth worldwide [13, 29, 30]. However, the agreement and discrepancies between the outputs of the HUI3 and SF-6D instruments have not been evaluated in head-to-head comparisons in a sample of VP or VLBW individuals.

This constrains efforts to enhance comparability and standardization of findings across different VP/VLBW studies, as well as reduces transparency and reproducibility of outcomes research in this area. It is important to determine the most appropriate HRQoL instruments for individuals born VP/VLBW because preterm birth and low birthweight represent a growing public health concern. Increasing VP/VLBW rates coupled with improvements in survival rates place increased pressures on healthcare budgets worldwide [13, 29, 30]. At the same time, the evidence regarding agreement between these measures in general population samples is opaque.

The first study that described (dis)agreement between the outputs of the HUI3 and SF-6D was published over twenty years ago [3]^1^; however, subsequent studies struggled to provide conclusive evidence and explain the source(s) of (dis)agreement. One explanation is that existing studies generally have not evaluated concurrent agreement of the HUI3 and SF-6D instruments in general population samples [31–39], since most studies recruited participants with specific conditions within clinical settings, such as tertiary care [31–39] or primary care [35]. Thus, existing findings may not be generalizable across general population contexts. To our knowledge, only one study has assessed levels of agreement between the HUI3 and SF-6D measures [40] among healthy individuals. However, given the overall study design employed, the evidence related to levels of agreement of the HUI3 and SF-6D measures within the general population is not conclusive [1]. Furthermore, results from other studies were based on patients recruited into clinical trials [31, 32, 38], which are prone to experimental design limitations [41, 42]. Finally, the majority of studies that assessed agreement between the outputs of the HUI3 and SF-6D were limited to one country or geographic region [31–39] and thus may have limited external validity.

Previous research called for comparative evaluations of the HUI3 and SF-6D measures across a diverse range of health conditions [43, 44]. Furthermore, research has advocated new comparative evaluation studies that use larger samples by maximizing their power and enhancing comparability when data across multiple cohort studies are combined [44]. To overcome the limitations associated with analyses restricted to a specific disease or disorder, conducted within limited clinical settings or within a single geographical region, the use of individual patient data analysis (IPD) consolidated over several geographically diverse cohorts offers advantages. This study uses IPD from European and Australian multi-site collaborative cohorts to inform the choice of the HUI3 and/or SF-6D measures for research studies that consider the consequences of VP/VLBW in adulthood as well as informs the cost-effectiveness of preventive or treatment interventions related to VP/VLBW status.

This study has the following aims: (a) to examine the agreement between the outputs of the HUI3 and SF-6D measures among adults born VP/VLBW and controls and to explain the sources of disagreement between instruments and (b) to provide useful information for the selection of preference-based HRQoL instruments for trials or research studies that ascertain the long-term consequences of VP/VLBW and birth at term or with normal birthweight.

## Methods

### Data

The following criteria have been utilized to identify relevant prospective cohorts: (1) have used two distinctive preference-based measures to assess HRQoL in adulthood (defined as ≥ 18 years [45]) amongst individuals born VP/VLBW, (2) included a comparison control group of term-born and/or normal birthweight individuals, and (3) contributed data to the RECAP consortium (www.recappreterm.eu), a database of cohorts of individuals born VP/VLBW. Two different and recent systematic reviews of preference-based HRQoL outcomes following preterm birth or low birthweight had identified eligible cohorts [46, 47]. The following two prospective cohort studies met the study inclusion criteria: The Bavarian Longitudinal Study (BLS) [48] and The Victorian Infant Collaborative Study (VICS) [49]. These two studies were designed to assess the associations of VP/VLBW status with various health outcomes [50] as well as received country-specific ethical approvals, including participants’ written informed consent in adulthood.

Table 1 described the background eligibility criteria, age(s) at assessment, and the control groups for the BLS and VICS cohorts. Detailed descriptions of each participating cohort (the study’s population, methodology, types of data and variables) have been previously published [48, 49]. All variables of interest across BLS and VICS were harmonized, meaning that an identical set of definitions, scaling methods, and classification were applied to all variables across BLS and VICS cohorts.

**Table 1:** Background characteristics of cohorts

	BLS (Germany)				VICS (Australia)			
	HUI3 at 26 years		SF-6D at 26 years		HUI3 at 18 years		SF-6D at 18 years	
	VPT/VLBW	Controls	VP/VLBW	Controls	EP/ELBW	Controls	EP/ELBW	Controls
Number completing MAUI	231	224	231	226	186	137	180	143
Age at assessment Mean (SD)	26.3	26.3	26.3	26.3	17.9	18.1	18.0	18.1
	(0.68)	(0.69)	(0.69)	(0.70)	(0.78)	(0.88)	(0.79)	(0.87)
GA at birth, Mean (SD)	30.6	39.7	30.6	39.7	26.7	39.0	26.7	39.2
	(2.19)	(1.18)	(2.18)	(1.18)	(2.10)	(1.43)	(2.10)	(1.44)
Birth weight, Mean (SD)	1330	3360	1330	3362	887	3419	886	3422
	(320)	(448)	(319)	(447)	(155)	(468)	(155)	(463)
Sex, n (%) male	125	105	124	105	84	60	82	60
	(54.1)	(46.9)	(53.7)	(46.5)	(45.2)	(43.8)	(45.6)	(42.0)

Study name	Bavarian longitudinal study	Victorian infant collaborative study
Birth Year	1985–1986	1991–1992
Eligibility Criteria VP/VPBW	VPT\VLBW (GA < 32wk or BW < 1500 g)	EPT\ELBW (GA < 28wk or BW < 1000 g)
Controls	Recruited in the same obstetric hospitals	Normal birth weight, contemporaneously recruited

*MAUI* multi-attribute utility instrument, *VP* very preterm (< 32 weeks GA), *VLBW* very low birth weight (< 1500-g birth weight), *EP* extremely preterm (< 26-week GA for EPICure and < 28-week GA for VICS), *ELBW* extremely low birth weight (< 1000 - birth weight), *SD* standard deviation

### Outcome measures

Participants’ perceptions of their HRQoL were assessed using both the HUI3 and SF-12 [48, 49]. Study participants completed the unedited Health Utilities Index 15-item questionnaire for usual health status assessment, which was obtained from the Health Utilities Index developers and covers the HUI3 health status classification system. The HUI3 was developed to describe HRQoL in general population and clinical contexts and consists of eight attributes: ambulation, dexterity, cognition, vision, hearing, speech, emotion, and pain [51–53]. Within each attribute, the levels of function were scored on a 5- or 6-point scale ranging from optimal function to severe impairment. Responses within each of the eight attributes can be valued as single attribute utility (SAU) scores on a scale ranging from 0 and 1 [51]. Responses within each of the eight attributes can also be mapped onto an eight-attribute health status vector. Algorithms reflecting the preferences of the general public for the HUI3 health states can be used to convert responses to the measure’s eight attributes into multiplicative multi-attribute utility scores. The Canadian algorithms [51–54] were applied in both cohorts, reflecting the preferences of 504 adults in the general population who were living in the city of Hamilton, Ontario, and who had previously been asked to value selected HUI3 health states using both visual analogue scaling and standard gamble techniques. HUI3 multi-attribute utility scores are valued on a cardinal scale ranging between -0.36 and 1.0, with -0.36 representing the worst possible HUI3 health state, 0.0 representing dead, and 1.0 representing full health [53, 54].

The SF-12 includes 12 of the 36 items contained within the SF-36. These have an identical dimension structure [55], and for each dimension, item responses are mapped onto a 0 to 100 scale. Responses to the SF-12 items were converted [56] into SF-6D multi-attribute utility scores using the UK SF-6D utility algorithms [55]. The SF-6D algorithms reduce the eight dimensions of the SF-36/12 to six by merging role limitations due to emotional and physical problems and eliminating general health perceptions. SF-6D multi-attribute utility scores are valued on a cardinal scale ranging between 0 and 1.0, with 0 representing dead and 1.0 representing full health [55]. For the SF-6D, only two out of six dimensions (physical functioning, role limitations) reflect physical aspects of health, while other dimensions (social functioning, pain, mental health, vitality, and emotional) relates to non-physical aspects of health. By contrast, most HUI3 attributes reflect the physical health of the individual (vision, hearing, speech, ambulation, and dexterity).

We used the following outcome variables of interest: HUI3 and SF-6D multi-attribute utility scores and the difference between HUI3 and SF-6D utility scores. The minimum clinically important difference in multi-attribute utility score is considered to be 0.03 for the HUI3 [57] and 0.04 for SF-6D [58, 59].

### Empirical analyses

We combined IPD across the BLS and VICS cohorts. To identify whether our assessments of agreement between the HUI3 and SF-6D measures should be disentangled by birth status, we initially estimated the association between VP/VLBW status and HRQoL in adulthood using one-stage IPD analysis, which could be implemented either using fixed or random effects [60]. Fixed effects models were used because individuals born VP/VLBW and controls were enrolled across distinct geographical regions and time frames. This implies the presence of systematic differences across the BLS and VICS cohorts. However, we also utilized random effects as a robustness check. Models were adjusted for age and sex of the participants, mode of delivery (cesarean section vs vaginal delivery), and number of days in hospital after birth, as well as for the harmonized socio-demographic/socio-economic variables: maternal education level at birth or during childhood and maternal ethnicity.

We computed means, standard deviations, and t tests for unequal variances, medians, and Kruskal–Wallis tests to assess differences in agreement between HUI3 and SF-6D multi-attribute utility scores within VP/VLBW individuals, controls, and the combined sample. To identify statistically significant predictors that explain observed differences between HUI3 and SF-6D multi-attribute scores on covariates, we used generalized mixed models in a one-step approach. Models were estimated using multivariate linear fixed effects.

Furthermore, agreement between the HUI3 and SF-6D multi-attribute utility scores was investigated using the intra-class correlation measures and Bland–Altman plots. The analysis was performed for VP/VLBW individuals and controls separately as well as for the combined sample. An intra-class correlation coefficient less than 0.75 is indicative of moderate agreement, while an intra-class correlation coefficient greater than 0.75 indicates good agreement [60, 61]. Bland–Altman plots display the mean $\left( {\frac{HUI3 + SF - 6D}{2}} \right)$ overall scores and the difference (HUI3-SF-6D) against each other. A line of mean difference estimates systematic difference between the two instruments, with limits of agreement estimated as the mean difference plus/minus 1*.*96 standard deviation of the mean difference. Limits of agreement (LoA) reflect the expected range in which 95% of observed differences would lie, with wider limits of agreement indicating poorer agreement [62]. Good concordance between the HUI3 and SF-6D would show a mean difference close to zero with ≤ 5% of scatter points lying outside the limits of agreement.

Analyses were performed using STATA version 17 and p-values of 0.05 or less were considered statistically significant.

## Results

### Baseline characteristics of prospective cohort studies

Table 1 displays baseline characteristics of the participants of the BLS and VICS cohorts. Years of birth ranged from 1985 to 1986 for BLS and 1991–1992 for VICS. Pooled data consisted of 778 HUI3 assessments (417 VP/VLBW individuals and 361 controls) and 780 SF-6D assessments (411 VP/VLBW and 369 controls). The mean age at assessment was 18 years for VICS participants and 26.3 years for BLS participants. Table 2 shows the characteristics of VP/VLBW individuals and controls with non-missing HUI3 and SF-6D multi-attribute utility scores. Within the meta-cohort no statistically significant differences were found by birth status across the following characteristics: age, sex of the participants, maternal education level at birth or during childhood, and maternal ethnicity.

**Table 2:** Characteristics of VP/VLBW individuals and controls within HUI3 and SF-6D Meta-cohorts

Meta-cohort (BLS & VICS)	VP/VLBW	Controls	*p* value	Missings/*N* (Pct)
N (%)	558 (53.2)	491 (46.8)		544/1593 (34.15)
Age at QoL assessment, mean (SD)	22.65 (4.32)	23.00 (4.12)	0.24	742/1593 (46.58)
Child sex, *N* (%)				
Male	276 (49.5)	233 (47.5)		
Female	282 (50.5)	258 (52.5)	0.52	1/1593 (0.06)
Gestational age (weeks), mean (SD)	28.51 (2.84)	39.41 (1.33)	< 0.001	1/1593 (0.06)
Birth weight (grams), mean (SD)	1090 (328.43)	3373 (442)	< 0.001	1/1593 (0.06)
Maternal age at birth (years), mean (SD)	28.68 (5.31)	29.14 (4.93)	0.15	8/1593 (0.50)
Mat educ at birth or childhood, *N* (%)				
Low level (equivalent to ISCED 0 to 2)	138 (33.0)	127 (36.7)		
Medium level (equivalent to ISCED 3 to 5)	221 (52.9)	146 (42.2)		
High level (equivalent to ISCED 6 to 8)	59 (14.1)	73 (21.1)	0.01	462/1593 (29.00)
Maternal ethnicity, *N* (%)				
Caucasian	493 (92.1)	445 (92.9)		
Non-Caucasian	42 (7.9)	34 (7.1)	0.65	39/1593 (2.45)
HUI3-MAU score, mean (SD)	0.85 (0.19)	0.89 (0.15)	< 0.001	815/1593 (51.16)
SF-6D MAU score, mean (SD)	0.83 (0.12)	0.83 (0.10)	0.23	813/1593 (51.04)

Table 2 reports characteristics for VP/VLBW and controls which had non-missing HUI3 or SF-6D Multi-Attribute Utility Scores

*Mat Educ* maternal education, *ISCED* International Standard Classification of Education. *SD* Standard deviation. When proportions are reported *p* value is based on Fisher’s exact test for equality of proportions. When means are reported the *p* value is based on a *t* test for unequal variances. Pct reports the percent of missing values.

### Relationship between VP/VLBW status and HRQoL using HUI3 vs SF-6D

Using a one-stage IPD meta-analysis, to identify whether our assessments of agreement between the HUI3 and SF-6D measures should be disentangled by VP/VLBW status, we initially estimated the association between VP/VLBW status and HRQoL in adulthood. The adjusted impact of VP/VLBW status on the HUI3 multi-attribute utility score was -0.04 (95% CI − 0.06, − 0.01) with no significant impact on the SF-6D multi-attribute utility score (Table 3). To understand the sources of identified differences we present the additional evidence in Online Appendix A (Tables A.1, A.2). We utilized random effects models and reported results in Online Appendix B. Further evidence on the association between VP/VLBW status and HRQoL in adulthood using HUI3 and SF-6D can be found in a recent study [63].

**Table 3:** One-stage IPD meta-analyses: Impact of preterm birth on HUI3-MAU score and SF-6DMAU score all cohorts combined

	Health utilities index mark 3 MAU score						Short form 6D MAU score					
	Unadjusted	95%CI	*p* value/*N*	Adjusted	95%CI	*p* value/*N*	Unadjusted	95%CI	*p* value/*N*	Adjusted	95%CI	*p* value/*N*
*βVPT/VLBW*	− 0.04	[− 0.06, − 0.02]	0.01/778	− 0.04	[− 0.06, − 0.01]	0.01/615	− 0.01	[− 0.02, 0.01]	0.45/780	− 0.01	[− 0.03, 0.00]	0.18/617
	(0.01)			(0.01)			(0.01)			(0.01)		
Mode of delivery				− 0.00	[− 0.03, 0.03]	0.91				− 0.00	[− 0.03, 0.02]	0.52
				(0.01)						(0.01)		
Hosp days				− 0.00	[− 0.00, 0.00]	0.728				− 0.00	[− 0.00, 0.00]	0.55
				(0.00)						(0.00)		
Sex (female)				− 0.02	[− 0.05, 0.01]	0.99				− 0.03	[− 0.05, − 0.02]	< 0.001
				(0.01)						(0.01)		
Mat educ (medium vs low)				0.00	[− 0.03, 0.02]	0.07				0.01	[− 0.01, 0.03]	0.22
				(0.01)						(0.00)		
Mat educ (high vs low)				0.02	[− 0.00, 0.06]	0.09				0.01	[− 0.01, 0.03]	0.34
				(0.01)						(0.00)		
Age at assessment				− 0.01	[− 0.02, 0.01]	0.05				− 0.00	[− 0.02, 0.01]	0.52
				(0.00)						(0.00)		
Cohort effects included	No			Yes			No			Yes		

Method: Linear Fixed Effects Models. HUI3-MAU and SF-6D column results are based on the following cohorts: BLS at 26 and VICS at 18. Mode of Delivery is an indicator for Cesarean section. Hosp. Days—number of days in hospital after birth. All models controlled for cohorts’ fixed effects. Robust standard errors in round parentheses

### Comparison of HRQoL assessed by the HUI3 and SF-6D

Table 4 displays descriptive and inferential statistics for HUI3 and SF-6D multi-attribute utility scores for each group considered. Mean and median estimates for HUI3 multi-attribute utility scores were consistently higher compared with their respective SF-6D values. All differences were clinically [57–59] and statistically significant within the meta-cohort across all groups considered (*p* < 0*.*01). Table 5 shows the estimates from regressing differences between HUI3 and SF-6D multi-attribute scores on covariates. The evidence suggests that none of the variables considered was a statistically significant predictor of observed differences between multi-attribute scores.

**Table 4:** HUI3 and SF-6D utility scores, differences between scores and quantification of agreement by VP/VLBW, controls, and combined sample

Cohorts	VP/VLBW	Controls	Combined	*p* value
BLS cohort				
* N* (%)	259 (53.1)	229 (46.9)	488 (100.0)	
HUI3-MAUI, mean (SD)	0.85 (0.18)	0.89 (0.14)	0.87 (0.16)	0.01
SF-6D MAUI, mean (SD)	0.83 (0.11)	0.84 (0.09)	0.83 (0.10)	0.49
Median ∆, (min; max)	0.05 (− 0.75; 0.40)	0.07 (− 0.61; 0.37)	0.06 (− 0.75; 0.40)	0.02
Mean ∆, (95% CI)	0.02 (− 0.00; 0.04)	0.05 (0.03; 0.07)	0.04 (0.02; 0.05)	0.02
VICS cohort				
*N* (%)	299 (53.3)	262 (46.7)	561 (100.0)	
HUI3-MAUI, mean (SD)	0.86 (0.20)	0.90 (0.15)	0.88 (0.18)	0.08
SF-6D MAUI, mean (SD)	0.82 (0.13)	0.83 (0.11)	0.82 (0.12)	0.35
Median ∆, (min; max)	0.04 (0.02; 0.07)	0.07 (0.05; 0.10)	0.06 (0.04; 0.07)	0.16
Mean ∆, (95% CI)	0.08 (− 0.83; 0.49)	0.08 (− 0.58; 0.36)	0.08 (− 0.83; 0.49)	0.10
BLS and VICS cohorts				
* N* (%)	558 (53.2)	491 (46.8)	1049 (100.0)	
HUI3-MAUI, mean (SD)	0.85 (0.19)	0.89 (0.15)	0.87 (0.17)	< 0.001
SF-6D MAUI, mean (SD)	0.83 (0.12)	0.83 (0.10)	0.83 (0.11)	0.23
Median ∆, (min; max)	0.06 (− 0.83; 0.49)	0.08 (− 0.61; 0.37)	0.07 (− 0.83; 0.49)	0.01
Mean ∆, (95% CI)	0.03 (0.01; 0.05)	0.06 (0.04; 0.07)	0.04 (0.03; 0.06)	0.01

∆ denotes the difference between HUI3 and SF-6D Multi-Attribute Utility Scores. *SD* standard deviation. When means are reported the *p* value is based on a paired *t* test. When medians are reported the *p*-value is based on a Kruskal–Wallis test

**Table 5:** HUI3 and SF-6D utility scores, differences between scores and quantification of agreement by VP/VLBW, and controls

	Outcome	∆				Outcome	∆			
	Coefficient	SE	Lower 95% CI	Upper 95% CI	*p* value	Coefficient	SE	Lower 95% CI	Upper 95% CI	*p* value
Age at assessment	− 0.00	0.00	− 0.01	0.00	0.84	− 0.00	0.01	− 0.02	0.02	0.94
Sex (female)	0.02	0.02	− 0.01	0.06	0.17	0.02	0.02	− 0.01	0.06	0.27
Mode of delivery	0.00	0.00	− 0.03	0.04	0.82	0.00	0.02	− 0.03	0.04	0.81
Hosp days	− 0.0	0.00	− 0.03	0.04	0.44	− 0.00	0.00	− 0.03	0.04	0.45
Mat educ (medium vs low)	0.01	0.02	− 0.04	0.05	0.78	0.01	0.02	− 0.03	0.05	0.87
Mat educ (high vs low)	0.05	0.03	− 0.01	0.11	0.09	0.05	0.03	− 0.01	0.10	0.14
Cohort effects included	No					Yes				

Linear Fixed Effects Models. The outcome variable is ∆ which denotes the difference between HUI3 and SF-6D Multi-Attribute Utility Scores. Models were adjusted for age and sex of the participants, mode of delivery (Cesarean section vs vaginal delivery), and number of days in hospital after birth, as well as for the harmonized socio-economic variables: maternal education level at birth or during childhood and maternal ethnicity

*SE* Robust standard error, *CI* confidence interval

The correlation coefficient (*ρ*) between HUI3 and SF-6D multi-attribute utility scores for the BLS and VICS cohorts was computed. The evidence showed that *ρ* between the two multi-attribute utility scores within VICS was 0.45 (*ρ* = 0.51 for VP/VLBW individuals and *ρ* = 0.31 for controls), which was higher compared with *ρ* = 0.35 within the BLS cohort (*ρ* = 0.37 for VP/VLBW individuals and *ρ* = 0.33 for controls). Within the meta-cohort, the ICC was 0.40 for the VP/VLBW sample, 0.29 for the controls, and 0.36 for the combined sample. Overall, the evidence suggests that the HUI3 and SF-6D multi-attribute scores had moderate or low correlation.

**Fig. 1:**
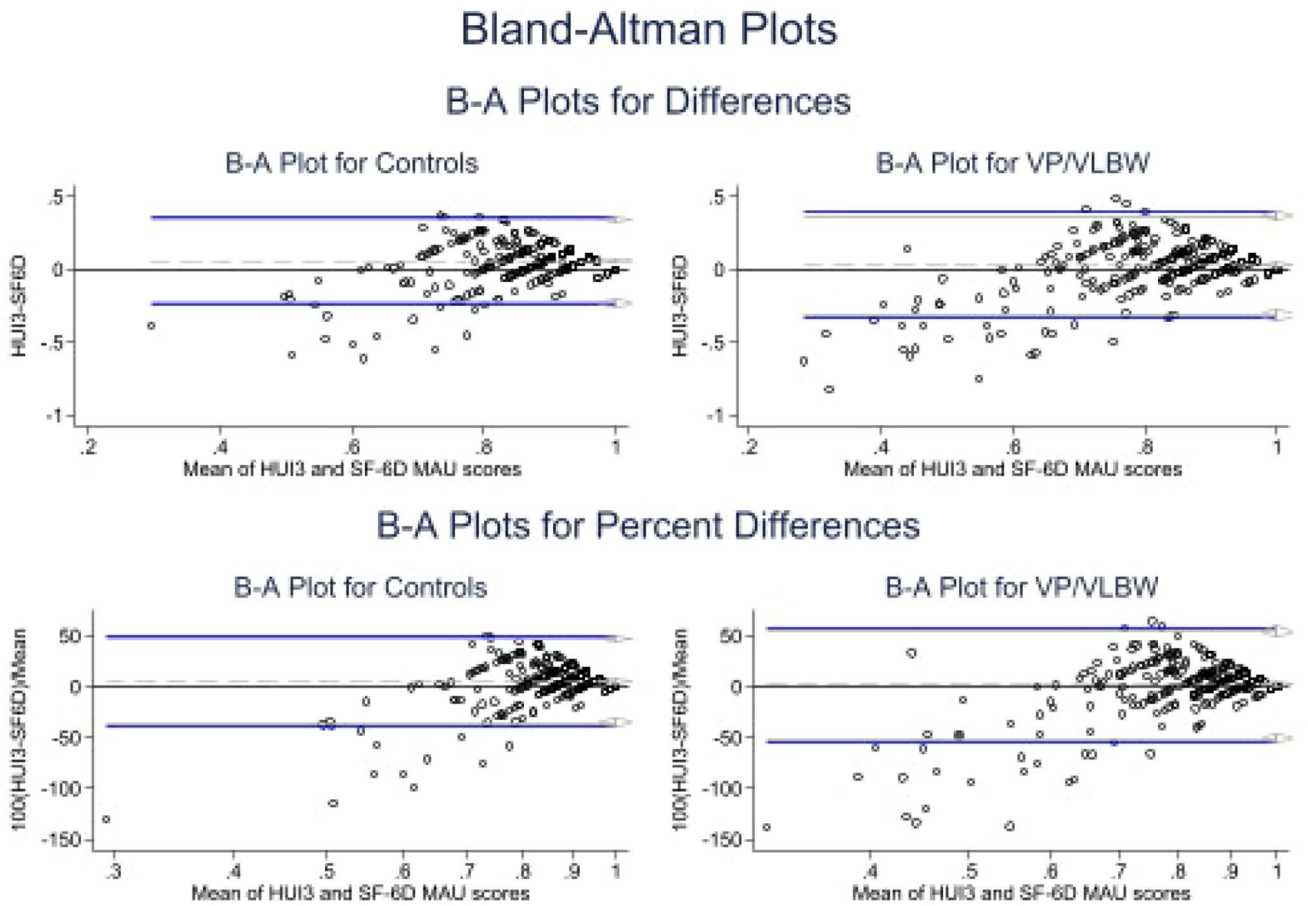
The Bland–Altman plots by VP/VLBW status

**Fig. 2:**
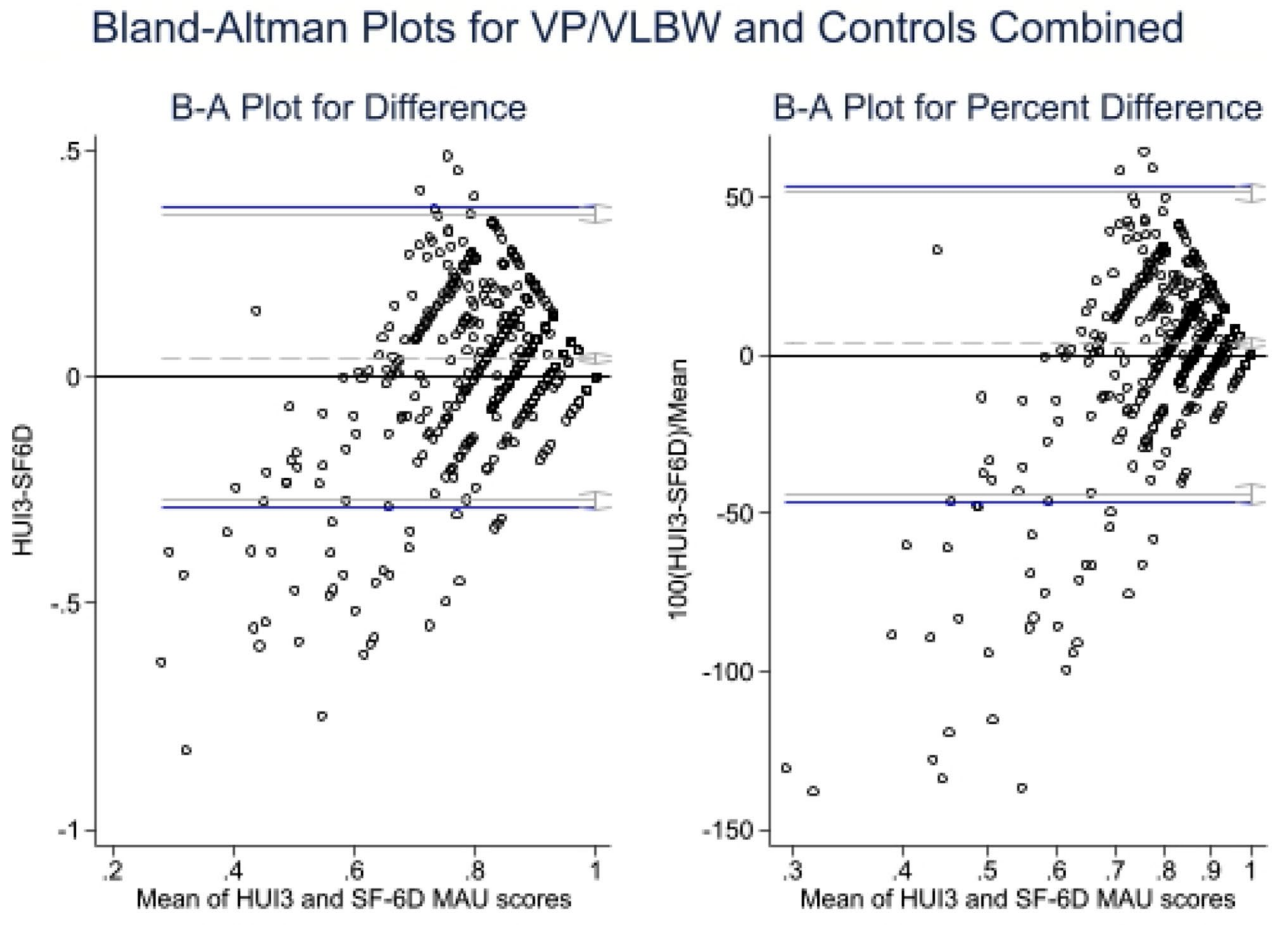
The Bland–Altman plots for VP/VLBW and controls combined

The Bland–Altman plots were constructed by birth status (see Fig. 1) and showed a mean difference of 0.06 (95% CI 0.04, 0.07), i.e., HUI3 multi-attribute utility scores for controls were higher than the SF-6D multi-attribute utility scores for controls. The mean difference for VP/VLBW individuals was 0.03 (95% CI 0.01, 0.05), meaning that HUI3 multi-attribute utility scores were higher than SF-6D multi-attribute utility scores in this group. In the Bland–Altman plot (Fig. 1), the data points deviate widely from the agreement line at low levels of mean utility and the relationship between the difference in HUI3 and SF-6D utilities shifts in magnitude but not in direction. The same pattern is observed by combining the VP/VLBW sample with controls (Fig. 2), generating a mean difference between the paired observations of 0.04 (95% CI 0.03, 0.06). Notably, in all groups considered, the Bland–Altman plots showed a funneling effect with stronger agreement as the mean overall utility score approached 1.0. However, in the Bland–Altman plots, the 95% LoA ranged from − 0.30 to 0.37 within the VP/VLBW sample, − 0.22 to 0.34 within controls, and − 0.27 to 0.36 within the combined sample. Most importantly, in all three groups considered (VP/VLBW, controls, and the combined sample), the 95% agreement differences were far wider than the clinically meaningful differences postulated for the HUI3 and SF-6D.

## Discussion

This study provides the first comparative evaluation of the HUI3 and SF-6D among adults born VP/VLBW and normal birthweight or term born controls. The results show a considerable degree of disagreement between the two sets of multi-attribute utility scores, consistent with previous reports for specific diseases [31, 33, 37, 40]. The patterns underlying differences vary, however, in a number of important aspects when compared with previous research. Our results identified less agreement compared with previous comparative evaluations of the HUI3 and SF-6D measures. Interestingly, our study found that agreement between the HUI3 and SF-6D measures was weaker in term-born or normal birthweight controls compared with VP/VLBW individuals.

Overall, the HUI3 and SF-6D measures disagree substantially because VP/VLBW status was found to be associated with minimal important decrements in utility score when health status was ascertained with the HUI3 and not the SF-6D. Furthermore, results show discordance between the outputs of the HUI3 and SF-6D in VP/VLBW individuals, controls, and the combined sample. This implies that the HUI3 and SF-6D each provide unique information on different aspects of health status across the groups considered and suggests that the HUI3 better captures preterm-induced changes to HRQoL in adulthood.

The evidence consistently demonstrates that the HUI3 and SF-6D instruments are not interchangeable for use in cost-utility based decision-making for interventions that target adults born VP/VLBW [64, 65]. Because our study also investigated concordance between the HUI3 and SF-6D in term-born or normal birthweight controls, the findings imply that the measures might also not be interchangeable for use in more general population samples.

Furthermore, given the evidence provided in this study regarding level of agreement between the HUI3 and SF-6D measures overall, our findings imply that studies focused on capturing the physical and cognitive effects of interventions should employ the HUI3 as a primary instrument, with the SF-6D as a potential supplementary measure. Our study implies that the HUI3 may be preferred to the SF-6D for studies designed at quantifying physical and cognitive aspects of health particularly since for SF-6D, only two out of six dimensions (physical functioning, role limitations) reflect physical aspects of health, while other dimensions (social functioning, pain, mental health, vitality, and emotional) relates to non-physical aspects of health. However, most HUI3 attributes reflect the physical health of the individual (vision, hearing, speech, ambulation, and dexterity). Prioritization of a preffered multi-attribute utility measure might increase the value of research design and potentially reduce unnecessary research costs related to primary data collection. Our results indicate that the HUI3 and SF-6D instruments are not interchangeable for use in clinical, population research, and cost-effectiveness based decision-making that considers the long-term consequences of VP/VLBW status [64, 65].

Our overall results are consistent with the differences in the HUI3 and SF-6D descriptive systems. Specifically, given that the HUI3 explicitly asks about a person’s vision, dexterity, ambulation, and cognition, while SF-6D does not, it is perhaps expected that VP/VLBW individuals, who are known to have impaired outcomes associated with these attributes [24–28], have lower levels of utility according to the HUI3 than according to the SF-6D. The evidence shows that discrepancies in the health descriptive systems of the HUI3 and SF-6D instruments may drive the differences in multi-attribute utility scores of VP/VLBW individuals and controls in adulthood. Our study demonstrates that variation in the descriptive systems of the measures is likely to be a major contributory factor to variation in the utility scores. Results of this research corroborate the conclusions of a study that analyzed patients in several disease areas and found that the EQ-5D, SF-6D, HUI3, 15D, QWB, and AQoL-8D instruments measure related but different constructs [44]. Also, the study concluded that the instruments differ in their relationship to different health dimensions, and the differences are primarily the result of the instruments’ descriptive systems.

Our study advances the literature because we provide clear evidence that differences in descriptive systems explain, at least in part, disagreement found between the outputs of the HUI3 and SF-6D measures. The evidence shows that the discordance between the outputs is observed within both adults born VP/VLBW and controls. However, differences related to HUI3 and SF-6D valuation protocols and utility ranges may also partly contribute to the differences in multi-attribute utility scores we document in this study. Furthermore, the study is the first in the literature to use a meta-analysis in this context combining data from two longitudinal prospective cohort studies.

This study does not infer that the HUI3 measure is generally preferable to SF-6D when health outcomes associated with clinical or public health interventions are ascertained. Rather, it provides insights for future research related to agreement between the HUI3 and SF-6D measures and suggests that the HUI3 classification system, unlike the SF-6D, is able to capture consequences of VP/VLBW status in adulthood, which is consistent with prior documented patterns reported in the disability literature [24–28]. We are not arguing against the use of the SF-6D or other preference-based HRQoL measures to investigate consequences of VP/VLBW status.

However, this study provides insight for stakeholders seeking to understand what instruments to use for comparative effectiveness research related to preterm birth and low birthweight. Further investigation is needed to understand the between-measure discrepancies attributable to descriptive classification systems for other measures, including the EQ-5D which is widely recommended in HTA guidelines [8, 17, 66–69] and other measures to inform the methodological debate and guide the selection of the most appropriate HRQoL instruments. Overall, the current study highlights the need to carefully consider the outcomes of interest and the characteristics being studied of the condition for an appropriate selection of HRQoL instrument.

### Strengths and limitations

The data structure made it possible to examine the agreement between measures within VP/VLBW individuals, normal birthweight or term born controls, and within the combined sample. We were able to assess the validity of the results by replicating the main finding across different populations, which strengthens the study’s conclusions and which had not been studied previously as far as we are aware. This is the major strength of this study. Furthermore, our study ascertained agreement between the outputs of the HUI3 and SF-6D measures using controls selected from the general populations in Germany and Australia. This implies that results of this study may be generalizable to populations from Germany and Australia.

Another strength of this study is that we were able to confirm VP/VLBW status in each participant due to the rigorous recruitment, data collection, and follow-up methods utilized by the participating cohorts, which also harmonized relevant socio-demographic factors. Furthermore, our study employed socioeconomically diverse samples of VP/VLBW individuals and controls. Finally, results of this study are not affected by biases associated with proxy parental reporting [70] because participating cohorts used self-reported HRQoL data.

It is important to note that the scoring algorithms for the HUI3 and SF-6D differ in certain respects. Thus, while our study shows that the utility differences we found are driven by the underlying concepts of health being measured, the methods employed are not able to measure the contributory effects of valuation protocols, i.e., differences in scoring algorithms. A further limitation is that our study included cohorts from only two countries. Thus, replication of this study with data from other countries, particularly low- or middle-income countries, would be a valuable contribution to the literature.

Our report did not include the EQ-5D in this comparative evaluation because no individual study that contributed to the RECAP platform assessed HRQoL using the EQ-5D. This is a limitation because a recent review identified that the EQ-5D is the most frequently recommended multi-attribute utility instrument in HTA guidelines [17]. Thus, our study is not able to provide comprehensive evidence regarding the most appropriate preference-based HRQoL measure to ascertain utility scores in adulthood for VP/VLBW individuals or for normal birthweight or term born controls. Comparing agreement of the EQ-5D, HUI3, and SF-6D for VP/VLBW individuals and normal birthweight or term born controls offers a fruitful direction for further investigation.

## Conclusion

The evidence from two longitudinal cohort studies conducted in Australia and Germany demonstrates poor agreement between the HUI3 and SF-6D in VP/VLBW individuals and normal birthweight or term born controls. It may be beneficial to use both the HUI3 and SF-6D instruments when evaluating health outcomes of interventions related to gestational age at birth and/or birthweight. However, studies focused on measuring physical or cognitive aspects of health will likely benefit from prioritizing the use of the HUI3 in order to better detect and quantify the effects of health interventions or assess outcomes.

## Supplementary Information

Below is the link to the electronic supplementary material.

Supplementary file1 (PDF 116 kb)

## Data Availability

Information regarding the data availability can be found at https://recap-preterm.eu/for-scientists/the-recap-preterm-cohort-platform/.

## Footnotes

1 This study recruited online participants who were registered with a panel company and who may have differed from the general population and narrowly defined as a healthy individual a respondent who indicated absence of a chronic disease using a controversial cut-off point (above 70 on an arbitrary numerical health scale, where 0 represented death and 100 represented best possible health). It excluded individuals who were too ill to complete the survey and did not harmonize the socio-economic factors associated with HRQoL across different countries. The authors were not able to verify the health status of participants or to control for clinical characteristics derived from medical records. Finally, given that participants self-selected into the study, its results might be biased.

## Code availability

The code used in this study is available from the authors upon reasonable request.
