# Supplementary material for: Comparative evaluation of the health utilities index mark 3 and the short form 6D: evidence from an individual participant data meta-analysis of very preterm and very low birthweight adults
Source: Qual Life Res. 2023 Jan 27;32(6):1703–16. doi: 10.1007/s11136-023-03344-x (PMC10172285; doi:10.1007/s11136-023-03344-x)
Supplement: Supplementary file 1 — Supplementary file1 (PDF 116 kb) [file 11136_2023_3344_MOESM1_ESM.pdf]

A Additional Evidence on VP/VLBW Association Across HUI3 and SF-6D Instruments

Table A.1: One–stage IPD Meta-analyses for HUI3 Single-Attribute Utility Scores Comparing VP/VLBW with Control Groups. Method: Linear Fixed Effects Models.

	Undjusted Model					Adjusted Model				
HUI3 Outcomes	$\beta_{VP/VPT}$	SE	Lower 95% CI	Upper 95% CI	**p-value**	$\beta_{VP/VPT}$	SE	Lower 95% CI	Upper 95% CI	**p-value**
HUI3-Vision Optimal Functioning	-0.09	0.03	-0.16	-0.02	0.01	-0.15	0.04	-0.22	-0.07	<0.001
HUI3-Vision SAU score	-0.01	0.00	-0.01	-0.00	<0.001	-0.01	0.00	-0.01	-0.00	0.01
HUI3-Hearing Optimal Functioning	-0.01	0.01	-0.03	0.01	0.46	-0.00	0.01	-0.02	0.02	0.81
HUI3-Hearing SAU score	-0.00	0.00	-0.00	0.00	0.79	0.00	0.00	-0.00	0.00	0.80
HUI3-Speech Optimal Functioning	-0.04	0.02	-0.09	0.00	0.06	-0.03	0.03	-0.08	0.02	0.19
HUI3-Speech SAU score	-0.00	0.00	-0.01	0.00	0.05	-0.00	0.00	-0.01	0.00	0.17
HUI3-Emotion Optimal Functioning	-0.06	0.03	-0.13	0.00	0.06	-0.08	0.04	-0.15	-0.00	0.04
HUI3-Emotion SAU score	-0.01	0.00	-0.01	0.00	0.08	-0.01	0.00	-0.01	0.00	0.15
HUI3-Pain Optimal Functioning	0.06	0.03	-0.01	0.12	0.07	0.04	0.04	-0.03	0.12	0.25
HUI3-Pain SAU score	0.00	0.00	-0.00	0.01	0.32	0.00	0.00	-0.00	0.01	0.30
HUI3-Ambulation Optimal Functioning	-0.03	0.01	-0.06	-0.01	<0.001	-0.04	0.01	-0.06	-0.02	<0.001
HUI3-Ambulation SAU score	-0.01	0.00	-0.02	-0.00	<0.001	-0.01	0.00	-0.02	-0.00	<0.001
HUI3-Dexterity Optimal Functioning	-0.05	0.01	-0.07	-0.03	<0.001	-0.06	0.01	-0.08	-0.03	<0.001
HUI3-Dexterity SAU score	-0.01	0.00	-0.01	-0.00	0.00	-0.01	0.00	-0.01	-0.00	<0.001
HUI3-Cognitive Functioning	-0.08	0.03	-0.15	-0.02	0.01	-0.08	0.04	-0.15	-0.01	0.03
HUI3-Cognitive SAU score	-0.01	0.00	-0.02	0.00	0.06	-0.01	0.01	-0.02	-0.00	0.05

Notes: Adjusted models controlled for: sex, maternal education, age at assessment and cohorts as fixed factors. Mat Educ - maternal education. SE – Robust Standard Error. CI - confidence interval. SAU – Single Attribute Utility Score.

Table A.2: One–stage IPD Meta-analyses for SF-6D Single-Attribute Utility Scores Comparing VP/VLBW with Control Groups. Method: Linear Fixed Effects Models.

	**Undjusted Model**					**Adjusted Model**				
SF-6D Outcomes	$\beta_{VP/VPT}$	SE	Lower 95% CI	Upper 95% CI	p-**value**	$\beta_{VP/VPT}$	SE	Lower 95% CI	Upper 95% CI	p-**value**
SF-6D Physical Optimal Functioning	-0.07	0.02	-0.11	-0.03	0.00	-0.06	0.02	-0.11	-0.02	0.01
SF-12 Physical Functioning score	-4.79	1.35	-7.44	-2.13	0.00	-4.78	1.56	-7.86	-1.71	<0.001
SF-6D Role Limitations Optimal Functioning	-0.04	0.03	-0.10	0.01	0.13	-0.05	0.03	-0.11	0.01	0.10
SF-12 Role Limitations score	-1.37	1.73	-4.77	2.04	0.43	0.67	1.92	-3.10	4.43	0.73
SF-6D Social Optimal Functioning	-0.02	0.03	-0.09	0.04	0.50	-0.05	0.04	-0.12	0.02	0.18
SF-12 Social Functioning score	-3.46	1.49	-6.37	-0.54	0.02	-4.13	1.54	-7.15	-1.10	0.01
SF-6D Pain Optimal Functioning	0.01	0.03	-0.04	0.07	0.66	0.00	0.03	-0.06	0.06	0.97
SF-12 Pain Functioning score	-0.50	1.09	-2.64	1.64	0.65	-0.69	1.22	-3.08	1.70	0.57
SF-6D Mental Health Optimal Functioning	0.04	0.03	-0.03	0.11	0.25	0.03	0.04	-0.05	0.10	0.46
SF-12 Mental Health score	-0.94	1.29	-3.48	1.60	0.47	-0.88	1.43	-3.69	1.93	0.54
SF-6D Vitality Optimal Functioning	0.06	0.02	0.02	0.10	0.00	0.04	0.02	-0.01	0.09	0.08
SF-12 Vitality score	-0.53	1.64	-3.74	2.68	0.75	-1.53	1.82	-5.10	2.04	0.40
SF-12 General Health score	-2.11	1.56	-5.18	0.96	0.18	-2.44	1.70	-5.77	0.90	0.15
SF-12 Role Emotional score	-2.28	2.12	-6.43	1.88	0.28	-2.81	2.17	-7.08	1.45	0.20

Notes: Adjusted models controlled for: sex, maternal education, age at assessment and cohorts as fixed effects. SE – Robust Standard Error. CI - confidence interval.

Results from the one-stage meta-analysis presented in Table A.1 demonstrate that VP/VLBW status was associated with sub-optimal levels of function and lower SAU scores for the following HUI3 attributes: vision, emotion, ambulation, dexterity and cognition. Analogous estimates for the SF-6D meta-cohort are presented in Table A.2. Within the SF-6D cohort, VP/VLBW status was associated with sub-optimal physical functioning and with lower scores for the following SF-6D dimensions: physical and social functioning.

B One-stage IPD Meta-analysis: Random Effects Models

Table B.1: One-stage IPD meta-analyses: Adjusted Impact of Preterm Birth on HUI3-MAU Score and SF-6D-MAU Score all Cohorts Combined. Method: Random Effects Models.

**Outcome**	$\beta_{VPT/VLBW}$	**SE**	Lower 95%**CI**	Upper 95%**CI**	p-**value**
HUI3 MAU score	-0.03	0.03	-0.09	0.02	0.09
SF-6D MAU score	-0.02	0.02	-0.06	0.01	0.20

Notes: All models controlled for cohorts' random effects. Results are adjusted for: Mode of Delivery is an indicator for Cesarean section. Hosp. Days - number of days in hospital after birth. SE – Robust standard error. CI - confidence interval.
